# Catalytic Oxidation of Lignin in Solvent Systems for Production of Renewable Chemicals: A Review

**DOI:** 10.3390/polym9060240

**Published:** 2017-06-21

**Authors:** Chongbo Cheng, Jinzhi Wang, Dekui Shen, Jiangtao Xue, Sipian Guan, Sai Gu, Kai Hong Luo

**Affiliations:** 1 Key Lab of Thermal Energy Conversion and Control of MoE, Southeast University, Nanjing 210096, China; 220130473@seu.edu.cn (C.C.); 220160516@seu.edu.cn (J.W.); 2 Jiangsu Frontier Electric Power Technology Co., Ltd., Nanjing 211102, China; ludong901122@sina.com (J.X.); Parallel79@163.com (S.G.); 3 Department of Chemical and Process Engineering, Faculty of Engineering and Physical Sciences, University of Surrey, Surrey GU2 7XH, UK; sai.gu@surrey.ac.uk; 4 Department of Mechanical Engineering, University College London, London WC1E 7JE, UK; k.luo@ucl.ac.uk

**Keywords:** biomass, lignin depolymerization, oxidative solvolysis, catalysis

## Abstract

Lignin as the most abundant source of aromatic chemicals in nature has attracted a great deal of attention in both academia and industry. Solvolysis is one of the promising methods to convert lignin to a number of petroleum-based aromatic chemicals. The process involving the depolymerization of the lignin macromolecule and repolymerization of fragments is complicated influenced by heating methods, reaction conditions, presence of a catalyst and solvent systems. Recently, numerous investigations attempted unveiling the inherent mechanism of this process in order to promote the production of valuable aromatics. Oxidative solvolysis of lignin can produce a number of the functionalized monomeric or oligomeric chemicals. A number of research groups should be greatly appreciated with regard to their contributions on the following two concerns: (1) the cracking mechanism of inter-unit linkages during the oxidative solvolysis of lignin; and (2) the development of novel catalysts for oxidative solvolysis of lignin and their performance. Investigations on lignin oxidative solvolysis are extensively overviewed in this work, concerning the above issues and the way-forward for lignin refinery.

## 1. Introduction

Biomass is an abundant renewable feedstock for the production of fuels, chemicals, and energy. Nowadays, about 10% of the world’s primary energy is from biomass [1]. Biomass is primarily used to generate heat and power, and it stands as the fourth largest source of energy in the word (after oil, coal, and natural gas) [2]. Fuels produced from biomass-refinery processes are replacing the fossil-based fuels in the daily life [3]. However, the use of biomass components and derivatives as the raw feedstocks for the chemical industry is still in the fledging stages [4,5,6,7,8]. With the pressure of the price of fossil fuels and the high cost of biorefinery processes of biomass, more and more attention has been paid on the production of value-added chemicals from biomass.

Biomass consists of three valuable fractions: 38–50% cellulose composed of semi-crystalline polysaccharide, 23–32% hemicellulose composed of amorphous multicomponent polysaccharide, and 15–25% lignin composed of amorphous phenylpropanoid polymer [9,10]. Hemicellulose has important applications for biofuel production and for the generation of valuable chemical intermediates (e.g., furfural) [11]; cellulose can be decomposed into valuable products such as biofuels and platform chemicals (including levulinic and formic acids, gammavalerolactone and derived products) [12]; and lignin is the most underutilized fraction, only approximately 2% of the lignin available from the pulp and paper industry is commercially used while the remainder is burned as a low value fuel [13]. There is a general lack of efficient processes for the utilization of lignin regarded as the most important renewable source of aromatics on the planet [14]. It becomes highly desirable to develop efficient technologies for the conversion of lignin to aromatic chemicals [15,16,17].

In the past decades, several strategies have been employed for lignin valorization, such as pyrolysis and gasification [18,19,20,21,22,23]. Lignin valorization in solvent systems for the production of renewable aromatic chemicals has attracted ever-increasing attention during recent years. The methodology for lignin depolymerization (LDP) in solvent systems can be categorized as hydrolysis, hydrogenolysis, oxidation and a two-step LDP. Lange et al. [3] and Chatel et al. [24,25] have published reviews on catalytic oxidation for lignin valorization into value-added chemicals. Some relevant reviews on bond cleavage mechanisms during lignin depolymerization have been published [26] and the separation and purification processes in lignin depolymerization have been reviewed recently [27]. Catalysis is regarded as a key factor to fulfill the promise of lignin depolymerization to specific products. Catalytic (homogeneous/heterogeneous) conversion of lignin for production of aromatic chemicals has also been reviewed in recent years [1,28,29,30,31]. Lignin oxidative solvolysis has been widely applied in the bleaching process for pulping industry due to its economic feasibility [32,33]. Phenolic products with low molecular weight were detected as the primary products from the oxidative solvolysis of lignin [33]. Organometallic catalyzed oxidation, biomimetic oxidation and enzyme-based oxidation have been studied [3,34,35], aiming at the cleavage of the designated inter-unit linkages [27]. Fundamental research on understanding and controlling the oxidative process is essential for promoting the production of specific valuable compounds [33].

The chemical structure and morphological properties of lignin are briefly introduced in this work, regarding the typical inter-unit linkages and lignin-related model compounds. Catalytic oxidation of lignin and lignin model compounds in solvent systems are then comprehensively overviewed, concentrating on the cracking of the inter-unit linkages and the effect of catalyst and solvent on the production of renewable aromatic chemicals.

## 2. Chemical Structure of Lignin

**Figure 1:**
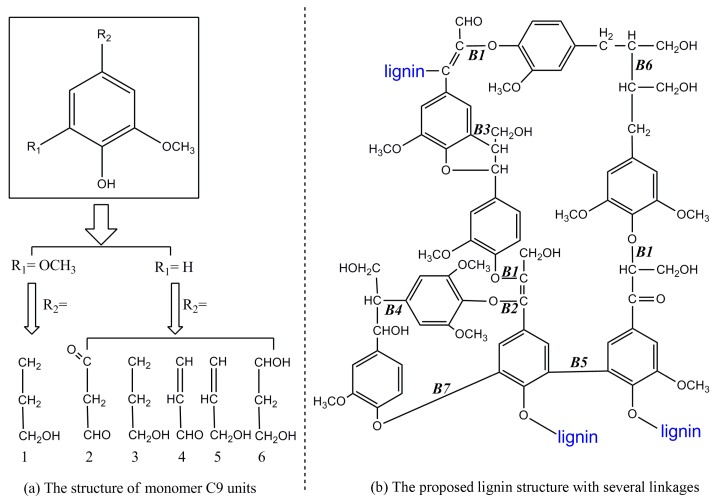
(**a**) Schematic of the structure of monomer C9 units; and (**b**) schematic of the proposed lignin structure with several linkages (B1: β-O-4, B2: α-O-4, B3: β-5, B4: β-1, B5: 5-5, B6: β-β, B7: 4-O-5) [44], reproduced with permission from Springer.

The roles of lignin in plants are to afford structural strength, protect the plants from degradation and enable the water conduction system connecting roots with leaves. Lignin is composed of *p*-hydroxyphenyl (H), guaiacyl (G) and syringyl (S) units linked by C–O or C–C bonds, which exhibit a complex three-dimensional amorphous structure. The content and chemical structure of lignin are primarily influenced by its sources and isolation methods. The chemical structure for lignin from different sources is different in terms of proportion of *p*-coumaryl-alcohol, coniferyl-alcohol, and sinapyl-alcohol (C9 unit) (Figure 1a). More than 95% of guaiacyl units (that derived from coniferyl alcohol) are estimated to be in softwood lignin, while the proportions of coniferyl-alcohols and sinapyl-alcohols are approximately equal in hardwood lignin. Herbaceous biomass (e.g., straw) lignin and woody lignin are of different chemical structures because of the extra content of *p*-hydroxyphenyl units and significant amounts of hydroxycinnamic acid (mainly *p*-coumaric acid). The proportions of *p*-coumaryl-, coniferyl- and sinapyl-alcohols in lignin depend on sources of lignin.

Lignin units are linked by either C–O or C–C bonds. More than two-thirds of the total linkages are C–O bonds while the others are C–C bonds in native lignin [1,36]. The carbon atoms in aromatic moieties are numbered as 1–6 and those in the aliphatic side chains of the units are labeled as α, β, and γ to distinguish various types of linkages between two structural units. The β-position is generally favored by coupling other units, leading to β-O-4, β-β, β-1 and β-5 becoming main linkages among the units of lignin [28,37,38,39]. The most common linkage in lignin is the β-O-4 ether bond, accounting for 45 to 48% in native lignin [40,41]. Other linkages include α-O-4, 4-O-5, 5-5, etc. The linkages in lignin are illustrated in Figure 1b, while the proportion of these linkages and the functional groups depends on the lignin sources and extraction methods [28,42,43].

**Figure 2:**
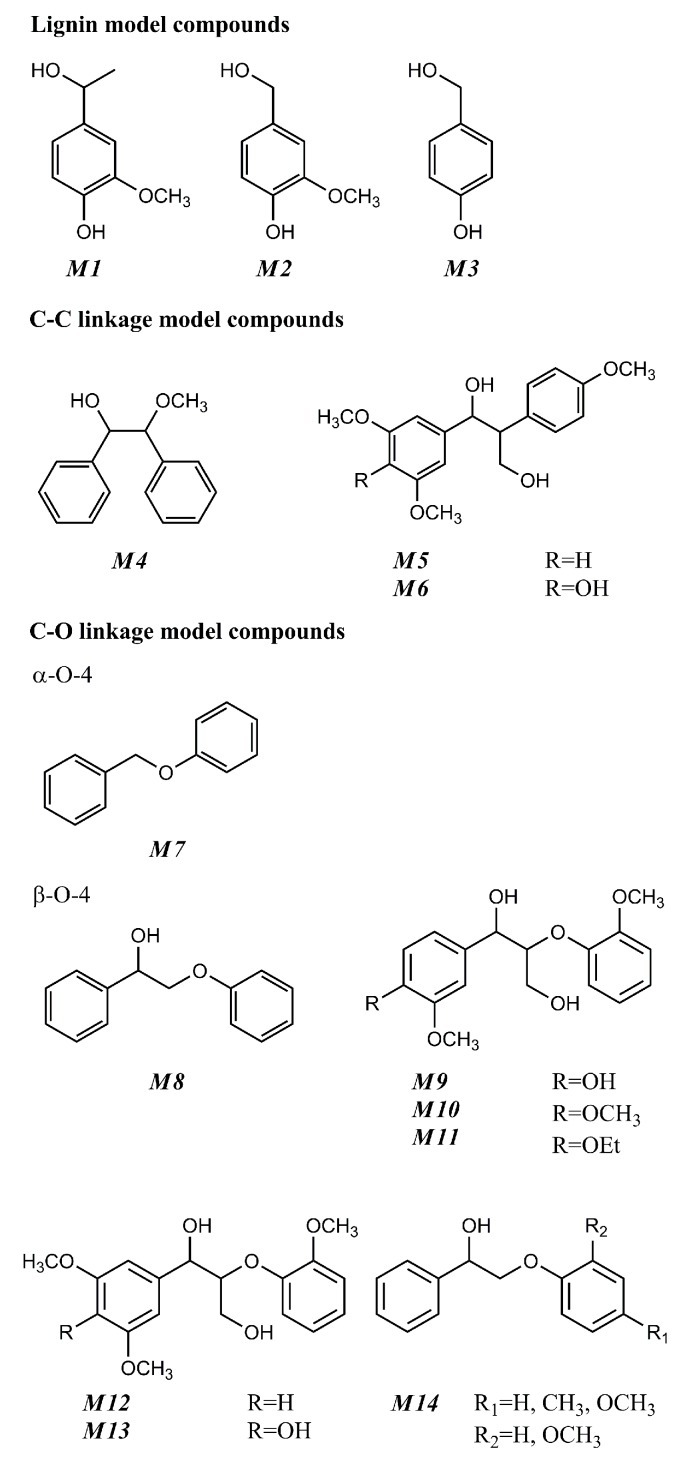
Lignin-related model compounds with different inter-unit linkages and functional groups.

Lignin-related model compounds are widely employed for better understanding of the cracking mechanism of the specific bonds in lignin. The most widely-reported model compounds and linkages containing aromatic or aliphatic ethers are shown in Figure 2. The synthetic methods for making β-O-4 model compounds [45], β-5 model compounds [46], β-1 model compounds [47,48] and β-β model compounds [49] have been reported in the literature. However, due to the lack of complexity of these models compared with lignin itself, the application of model compound in the development of selective LDP has been restricted [46]. Unlike a wide variety of trimer, tetramer, hexamer and polymer lignin model compounds containing only β-O-4 linkage, Ouyang et al. [50] synthesized a lignin model compound composed of three guaiacyl structural units linked by α-O-4 and β-O-4 ether bonds, via a three-step sequence method assisted with microwave irradiation. Sheldrake et al. [46] also reported a flexible, efficient and convergent preparation method for synthesizing model lignin hexamers and octamers linked by β-O-4, 5-5′ and β-5. These lignin model compounds can reflect some natural information of lignin, giving a better understanding of the inherent mechanism of lignin depolymerization processes. Other novel approaches for synthesizing the model compounds that are more accurate in mimicking characteristics of lignin are still required.

Solvolysis is a promising method for converting lignin to various valuable aromatic chemicals. The lignin macromolecule depolymerization and fragments repolymerization are involved in this process, which is conducted by using different catalysts, solvent systems, experimental conditions and even heating methods. The depolymerization of lignin to renewable aromatic chemicals is difficult due to the thermally stable, amorphous and complex structure of lignin. Lignin depolymerization often necessitates harsh conditions to break the stable structure of lignin. Under such conditions, the undesired repolymerization of fragments is favored, resulting in a decrease of product yield. The target linkages of lignin might be hard to access by heterogeneous catalysts because of the steric hindrance of the lignin amorphous backbone. Although the target linkages of lignin could access by homogeneous catalysts, it is difficult to separate homogeneous catalysts from the liquid products. The variability of a large number of substructural units in lignin would result in different catalytic reaction rates at different sites. A number of lignin sources from different isolation methods should be examined concerning the selection of feedstock for LDP.

### 2.1. Kraft Lignin

Kraft pulping is the predominant chemical pulping process around the world. This process is directed at temperatures in a range of 150–180 °C for around two hours and at high pH with the presence of considerable amounts of aqueous sulfide, sulfhydryl and polysulfide ions. Thus, the native lignin will undergo dramatic chemical and structural changes due to these severe conditions, which makes it difficult to be depolymerized to fragments in the subsequent processes. The percentage of lignin in the black liquor generated by kraft pulping ranges from 29 to 45% for cook of paper grade and 8 to 16% for cook of liner grade [51] and the percentage of kraft black liquor lignin extracted from hardwood is generally lower than that of softwood.

### 2.2. Sulfite Lignin

As kraft pulping becomes the dominant pulping process all around world, just about 10% of the pulp is produced by sulfite pulping process now [52]. Sulfite pulping is conducted under pH 2–12, depending on the cationic composition of the pulping liquor. Nearly all sulfite pulping processes are acidic, and calcium or magnesium (Ca^2+^/Mg^2+^) are used as the counterions, while, in higher pH environments, sodium or ammonium are usually used instead of Ca/Mg. Due to the sulfite presence, lignin is rendered soluble throughout the full range of pH; it cannot simply be isolated by pH adjustment. As mixtures with only 70–75% actually lignin, commercial sulfite lignin shows wide molecular weight profiles and variable grades of sulfonation, making it difficult to be applied in lignin depolymerization. The average molecular weight of sulfite lignin from softwood is notably higher that of the sulfite lignin from hardwood.

### 2.3. Organosolv Lignin

Organosolv lignin, derived from organosolv pulping process, is designed to dissolve lignin from plant cell walls by using a high proportion of an organic solvent (e.g., dioxane) in the cooking liquor [53]. Compared to kraft and sulfite pulping, organosolv pulping is a more environmentally benign pathway. Organosolv lignin reserves a large extent of its original structure in the form of original inter-unit linkages (e.g., β-O-4 linkage), which can be used as an appropriate lignin sample for subsequent conversion and valorization.

### 2.4. Pyrolytic Lignin

Pyrolytic lignin has unusual structural properties featured by its C8 basic unit skeleton and lower average molecular weight [54,55]. The average molecular weight of the pyrolytic lignin was reported to be within a range of 650–1300 Da [55]. Pyrolytic lignin is composed of some thermally ejected oligomers and the side chains in pyrolytic lignin are primarily in the form of inter-unit C–C linkages, leading to the difficulty of its application in LDP.

### 2.5. Dilute Acid Lignin

Diluted acids (e.g., H_2_SO_4_ and HCl) pulping have been applied in all kinds of lignocellulosic materials. Diluted acid pulping is generally conducted at a temperature of >160 °C with the presence of mineral acids as a catalyst. The C–O linkages as well as ester linkages are partially cracked during the diluted acid pulping process. This process may also undermine the three-dimensional amorphous structure in lignin and produce small lignin fragments with the increased proportion of hydroxyl groups [56,57]. Thus, lignin should be removed under benign conditions if it is considered as a feedstock for further chemical production.

### 2.6. Steam Explosion Lignin

The steam explosion pulping process is a fast processing method for lignocellulose, which releases single biomass components via steam impregnation under pressure and then the fast release of pressure [51]. For example, the disposal of wood or bagasse is conducted under high-pressure steam at a short contact time. More than 90% of lignin in hardwood can be recovered from the steam explosion pulping process. The properties of steam explosion lignin are similar to that of organosolv lignin, showing both a higher solubility and a lower molecular weight in an organic solvent. It is also composed of phenolic oligomers with 3 to 12 benzene rings. As a result, steam explosion lignin has potential for producing renewable chemicals through LDP.

## 3. Oxidative Solvolysis of Lignin

Oxidative solvolysis of lignin is a widely used and efficient strategy for producing multiple aromatic monomers. The oxidative solvolysis of lignin reaction includes the cracking of C–O bonds, C–C bonds in lignin. Aromatic aldehydes and carboxylic acids are the main products from oxidative solvolysis of lignin with different kinds of homogeneous catalysts. O_2_, H_2_O_2_, metal oxides and nitrobenzene have been considered as effective oxidation agents. In this section, the effect of catalyst and solvent on the production of valuable aromatic chemicals in the catalytic oxidation of lignin process will be discussed. Research on oxidative solvolysis of raw lignin (Kraft lignin, Organosolv lignin, etc.) to the aromatic products is summarized in Table 1.

### 3.1. Effects of Catalysts

Catalysts are involved in most cases of the oxidative depolymerization of lignin to produce particular products and even perform chemoselective oxidation of a particular linkage in lignin [77,78]. The catalysts widely used for the oxidative solvolysis of lignin can be divided into five groups: organometallic catalysts, metal-free-organic catalysts, acid/base catalysts, metal salts catalysts, and zeolite catalysts (heterogeneous catalyst).

#### 3.1.1. Organometallic Catalysts

**Figure 3:**
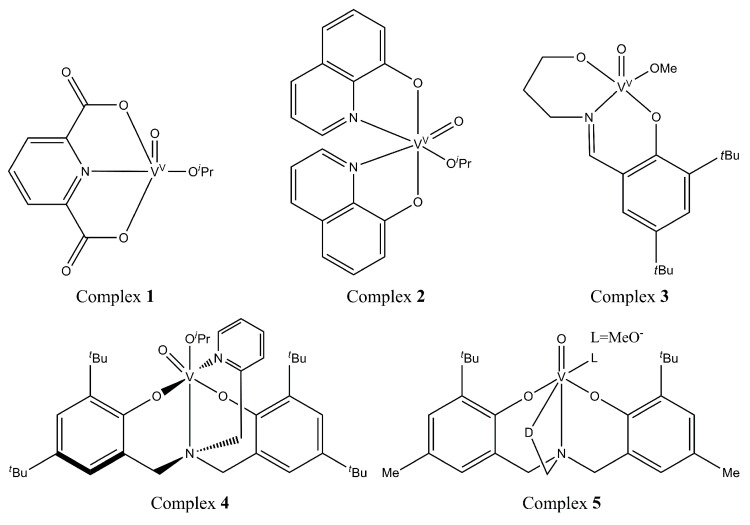
Schematics of the catalyst: vanadium Complexes **1**–**5**.

Several studies have reported on the oxidative solvolysis of raw lignin by using an organometallic catalyst. Chan et al. [58] demonstrated that a series of vanadium complexes bearing Schiff base ligands (e.g., Complex **3** in Figure 3) could be used to degrade organosolv lignin (extracted from Miscanthus giganteus using dioxane, acetone, and ethanol) into phenolic products in acetonitrile–THF or ethyl acetate–THF. After 24 h of reaction at 80 °C under air, vanillin, syringic acid and syringaldehyde were the most common degradation products, resulting from the guaiacyl and syringyl units of lignin, respectively (Table 1, Entry 1). Pretreatment and isolation methods could remarkably alter the structure of lignin and thus affect the reactivity of lignin toward catalysts. Then, vanadium Complexes **1**, **2** and **4** (Figure 3) and other organometallic catalysts such as 4-acetamido-TEMPO/acid (TEMPO = 2,2,6,6-tetramethylpiperidine-1-oxyl) and (salen)cobalt(II) were also used in the catalytic oxidation of organosolv lignin [59]. Though most catalysts oxidized the lignin extracts, bis(8-oxy-quinoline) oxovanadium and bis(phenolate)pyridine oxovanadium catalyst, Complex **2** and Complex **5** (Figure 3), respectively, exhibited the best performance for depolymerization/oxidation of organosolv lignin. After 18 h of reaction at 100 °C in 0.8 MPa of synthetic air (8% O_2_ in Ar), the best depolymerization performance was exhibited by Complex **2**, which gave the largest molecular weight (*M*_W_) shift and a considerable increase in the lower molecular weight products (Table 1, Entry 2). Iron complexes could also be suitable for oxidation of lignin. {Fe-DABCO} (DABCO = 1,4-diazabicyclo[2.2.2]-octane) catalyst was presented for the cleavage of β-O-4 linkages in lignin in the presence of H_2_O_2_ [60]. After 8 h of reaction at 100 °C, the mass maximum in the lignin sample had shifted from 3200 Da to around 2500 Da (Table 1, Entry 3). GPC analysis showed that the mass maximum had shifted to lower masses in all lignin samples, providing evidence for the formation of low molecular weight products. In addition, all of the β-O-4 linkages and resinol structures were degraded independent of the lignin pretreatment and isolation conditions.

Co(salen)/oxidant has been proven to be an efficient catalytic system for oxidation of phenolic and nonphenolic β-O-4 aryl ether lignin model compounds [79,80]. To improve complexes’ stability, selectivity, and recyclability, Co salen complexes were immobilized on graphene oxide (GO) (Co–GO) for the oxidation of lignin [61]. After 24 h of reaction at 80 °C in air, organosolv lignin was converted to vanillin (the main product), vanillic acid, 1-(4-hydroxy-3-methoxyphenyl) ethanol, and other monomeric phenolic compounds (Table 1, Entry 4). The oxidation of aliphatic groups resulted in the formation of carbonyl compounds, cleavage of β-O-4 bonds, demethylation of methoxy groups and formation of phenolic OH groups.

Recently, the first transition metal system using O_2_ as an oxidant for cleaving the resinol structure was reported [62]: An oxidative solvolysis of lignin with inexpensive transition-metal-containing hydrotalcites (HTc–Cu–V) or combinations of V(acac)_3_ (acac = acetylacetonate) and Cu(NO_3_)_2_·3H_2_O as catalysts. As the homogeneous copper and vanadium species from V(acac)_3_/Cu(NO_3_)_2_·3H_2_O and the leached HTc–Cu–V were most probably very similar in nature, after 40 h of reaction at 135 °C, the oxidative solvolysis of lignin afforded products of a mass of approximately 300 Da by using copper-vanadium double-layered hydrotalcites or combined V(acac)_3_ (acac = acetylacetonate) and Cu(NO_3_)_2_·3H_2_O as catalysts (Table 1, Entry 5). 

Methyltrioxo rhenium (MTO) acts as an active and efficient catalyst for the oxidation of phenolic and non-phenolic lignin model compounds representing the main bond patterns in native lignin resulting in the side-chain oxidation and aromatic ring cracking reaction [81]. Crestini et al. [63] developed immobilized MTO catalysts such as poly(4-vinylpyridine)/MTO and polystyrene/MTO as an easy recovery, reuse and low environmental impact catalyst, for the oxidation of lignin (Table 1, Entry 6). Different reaction pathways occurred between soluble MTO and immobilized MTO catalysts in the oxidative solvolysis of lignin: compared to homogeneous MTO, immobilized MTO catalysts with lower Lewis acidity might direct their reactivity toward aliphatic C–H insertion and Dakin-like reactions instead of oxidizing the aromatic ring. Consequently, these differences gave rise to the final product with a higher amount of free phenolic guaiacyl groups in the presence of immobilized MTO catalysts.

#### 3.1.2. Metal-Free-Organic Catalysts

Some metal-free-organic catalysts have been identified as effective catalysts for the oxidation of β-O-4 and α-O-4 types of lignin model compounds. Gao et al. [64] used nitrogen-containing graphene material (LCN) to convert birch wood lignin into depolymerized products in water and *tert*-butyl hydroperoxide solvent. LCN exhibited a similar catalytic behavior in the oxidation of lignin model compounds and birch wood lignin. The catalytic oxidation of benzylic C–H and C–OH bonds into carbonyl groups broke the C_α_–C_β_ and C_α_–O bonds, and further catalyzed the decomposition of some unstable aromatic compounds. Finally, 45.8 wt % bio-oil could be obtained from birch wood lignin after 24 h of reaction at 140 °C in the presence of 0.1 MPa O_2_ (Table 1, Entry 7).

#### 3.1.3. Acid/Base Catalysts

NaOH and KOH are utilized most frequently in the alkaline oxidation of lignin. Ouyang et al. [82] reported the oxidative degradation of soda lignin assisted by microwave on NaOH catalyst (pH = 11). Similarly, Wu et al. [83] reported the oxidative degradation of different lignin samples in KOH with H_2_O_2_ or K_2_S_2_O_8_ to methanol, formate, carbonate, and oxalate. Even at near-ambient temperatures (60 °C), >90% consumption of lignin was observed after 24 h. The microwave irradiation efficiently helped the degradation of lignin to produce high molecular weight compounds. The origin of the lignin, reaction temperature, oxidant concentration, and pH value also had impacts on the conversion efficiency. For example, higher reaction temperature and O_2_ concentration facilitated the yield of the final products but also gave rise to the repolymerization of lignin fragments. Maziero et al. [84] also reported that sugarcane bagasse lignin oxidized with 9.1% H_2_O_2_ (*m*/*v*) at pH = 13.3 had the highest fragmentation, oxidation degree and stability. The oxidation of alkali lignin was performed on NaOH catalyst (pH = 11) in the presence of O_2_ [65]. After 20 min of reaction, the maximum total yield of products (formic acid, acetic acid, succinic acid, oxalic acid, and glutaconic acid) of 44 wt % was obtained at 225 °C and 1 MPa O_2_ (Table 1, Entry 8).

Ouyang et al. [66] reported the oxidative solvolysis of lignin by using H_2_O_2_ as the oxidant and combination of CuO, Fe_2_(SO_4_)_3_ and NaOH as catalysts. Cu^2+^ could result in the removal of side chains from basic units of lignin forming some phenolic compounds while with OOH radicals generated by dissociation of H_2_O_2_, Fe^3+^ could form new reactive intermediates [85], facilitating oxidative solvolysis of lignin. The yield of monophenolic compounds with a higher amount of syringyl unit compounds (syringaldehyde, syringic acid, acetosyringone) reached 17.92 wt % in methanol/water (1:1 (*v*/*v*)) solvent at 150 °C after 1 h reaction (Table 1, Entry 9). Wood lignin from loblolly pine was oxidized by 1,10-phenanthroline and copper (II) sulfate pentahydrate and NaOH catalysts in methanol solvent [67]. Finally, 3.5 wt % vanillic acid and 12.6 wt % vanillin could be obtained after 24 h of reaction at 80 °C in the presence of 0.27 MPa O_2_ (Table 1, Entry 10).

#### 3.1.4. Metal Salt Catalysts

Polyoxometalates (POMs) consists of a number of metal oxygen cluster anions. They are soluble in both water and organic solvents, and its redox potential is high enough to oxidize lignin basic units yet low enough to be reoxidized by O_2_. A series of POMs are capable of selectively degrading the residual lignin and, in turn, fully converting residual lignin into CO_2_ and H_2_O by wet oxidation [86]. The POM treatment of lignin leads to a sharp reduction in the content of α-OH/β-O-4 inter-unit linkages, following demethylation. A high increase in carbonyl groups implies the loss of aromaticity in lignin, probably due to the conversion of aromatic rings to quinone moieties [86,87]. Voitl and Rohr [68] conducted the oxidative solvolysis of lignin by using aqueous POMs in the presence of alcohols for converting kraft lignin into chemicals. After 20 min of reaction, a total yield of products (vanillin and methyl vanillate) of 5.2 wt % could be obtained at 170 °C and 0.5 MPa O_2_ (Table 1, Entry 11). In a stirred batch reactor, vanillin (4.6 wt %) and methyl vanillate (4.2 wt %) could be obtained at 170 °C and 1.0 MPa O_2_ (Table 1, Entry 12) [69].

Transition metal salts are another kind of homogeneous catalysts for lignin oxidation, which have a wide range of cation redox potential. Werhan et al. [70] reported the acidic oxidation of kraft lignin with different transition metal salts (CuSO_4_, FeCl_3_, CuCl_2_, CoCl_2_). The maximum yield of vanillin gained was invariably higher for the transition metal salts than for the POMs. CoCl_2_ showed the best performance among the investigated catalysts with a maximum yield of 6.3 wt % (vanillin and methyl vanillate) at 170 °C and 1.0 MPa O_2_ (Table 1, Entry 13). In addition, the H_2_O_2_ mediated oxidative solvolysis of kraft lignin was also investigated in the presence of different metal salt catalysts (Fe_2_(SO_4_)_3_, Fe(NO_3_)·9H_2_O, Mn(SO_4_)·H_2_O, Bi_2_(SO_4_)_3_, Bi(NO_3_)_3_·5H_2_O, H_2_WO_4_, and Na_2_WO_4_·2H_2_O) in acetone/water with the use of ultrasound irradiation [71]. The Na_2_WO_4_·2H_2_O catalyst was the most efficient regarding the total yield of vanillin-based monomers (vanillin, acetovanillone, vanillic acid, and guaiacol) with a maximum yield of 0.51 wt % at 45 °C (Table 1, Entry 14). In addition, the use of ultrasound irradiation in this experiments results in high oxidative coupling of phenoxy radicals generated from LDP.

The metal/bromide catalyzed aerobic oxidation of alkylaromatic compounds in acetic acid solvent have shown to be a well-established method to produce aromatic carboxylic acids [88]. Thus, Partenheimer [72] investigated the aerobic oxidative solvolysis of lignin via Co/Mn/Zr/Br catalysts in acetic acid-water solvent. After 2 h of reaction at 180 °C in the presence of 13.8 MPa air, the highest yields of aromatic compounds with a total of 10.9 wt % could be obtained from organosolv lignin (Table 1, Entry 15).

In summary, most of these homogeneous systems lack selectivity for lignin, resulting in a relatively low yield of the target products. The separation and recyclability of homogeneous catalysts is still challenging for the catalytic oxidative solvolysis of lignin. Current research is focused on developing novel homogeneous catalysts and another kind of catalysts—heterogeneous catalysts—for the catalytic oxidative solvolysis of lignin.

#### 3.1.5. Heterogeneous Catalyst

Considering the limitations of homogeneous catalytic oxidative solvolysis of lignin, heterogeneous catalytic systems have been developed with regard to their advantages in achieving a relatively high yield of the product, combined with an easy separation and recyclability of the catalyst. Microwave assisted catalytic oxidation of organosolv lignin over lanthanum modified SBA-15 catalyst was investigated [73]. The catalytic oxidation with H_2_O_2_ led to different yields of aldehydes, and the respective acids and aceto derivatives. The highest yield of vanillin was 9.94 wt % and that of syringaldehyde was 15.66 wt % (Table 1, Entry 16). Then, the oxidative solvolysis of organosolv lignin in methanol using Pd/CeO_2_ catalyst was performed [74]. After 24 h of reaction at 185 °C under O_2_, several aromatic monomers including vanillin, guaiacol and 4-hydroxybenzaldehyde were identified and the yields of these monomers were 5.2, 0.87 and 2.4 wt %, respectively (Table 1, Entry 17). After that, Deng et al. [75,76] synthesized the perovskite-type LaMnO_3_ and LaCoO_3_ catalysts for catalytic wet aerobic oxidation of lignin. Both the perovskite-type oxides, LaMnO_3_ and LaCoO_3_, were efficient and recyclable heterogeneous catalysts for catalytic oxidative solvolysis of lignin. When LaMnO_3_ was used, the lignin conversion was 57.0% after 3 h at 120 °C in the presence of 0.5 MPa O_2_, and the maximum yields of vanillin, *p*-hydroxybenzyl aldehyde and syringaldehyde were 4.32 (30 min), 2.03 (120 min), and 9.33 wt % (30 min) respectively (Table 1, Entry 18). When LaCoO_3_ was used, the lignin conversion after 3.0 h of reaction increased 46.7%, and the maximum yield of vanillin, *p*-hydroxybenzyl aldehyde and syringaldehyde were 4.55 (60 min), 2.23 (120 min), and 9.99 wt % (50 min) respectively (Table 1, Entry 19).

It can be concluded that both homogeneous and heterogeneous catalysts for the oxidative solvolysis of lignin should be further developed due to the low selectivity and production for specific compounds.

### 3.2. Effects of Solvent System

The oxidative solvolysis of lignin has been conducted with the help of molecular O_2_ or aqueous H_2_O_2_ as oxidants. The oxidation of raw lignin as well as lignin model compounds using molecular O_2_ or aqueous H_2_O_2_ as oxidants is highly promising. It was reported that the removal of O_2_ in the system of H_2_O_2_ as an oxidant had no effect on the oxidation reactions [83]. The in-situ oxygen produced by the decomposition of H_2_O_2_ during the reaction might be more reactive than the external oxygen.

Solvent molecules could coordinate or dissociate to the vacant metal coordination sites in homogeneous catalysis [89]. Different solvents in the oxidative solvolysis of lignin result in a significant change of the yields of aromatic products. Chan et al. [58] conducted the oxidative solvolysis of organosolv lignin into phenolic products in acetonitrile and ethyl acetate. Both acetonitrile and ethyl acetate were optimal solvents, whereas THF had to be added to contribute to the total miscibility of all the reaction components. Ma et al. [90] reported the selective oxidative C–C bond cleavage of a lignin model compound over a vanadium catalyst in different solvents. In triethylamine, the C–H bond cleavage was preferred over the vanadium-catalyzed oxidation with O_2_, while C–C bond cleavage was dominant in acetic acid. It was believed that the coordination of the carboxylic group in acetic acid solvent to vanadium (V) catalyst was vital for C–C bond cleavage.

The oxidative solvolysis of lignin generates a number of radicals resulting in the repolymerization reaction to limit the production of aromatic compounds. Therefore, some research explored the addition of some radical scavengers in order to quench the reactive lignin fragments before repolymerization took place. The alcohol as a quenching agent could generate the radicals such as CH_3_O· or ·CH_3_. The role of those alcohols was investigated in the acidic degradation of lignin [68]. Methanol and ethanol might prevent the condensation through the competitive reaction with intermediate carbonium ions. Radical coupling of lignin fragments with CH_3_O· and ·CH_3_ produced from methanol occurred via acid-catalyzed formation of dimethyl ether (Equations (1) and (2)).

2CH_3_OH → CH_3_OCH_3_ + H_2_O (1)

CH_3_OCH_3_ → CH_3_O· + ·CH_3_ BDE = 84 kcal·mol^−1^ (2)

The effect of varied alcohol solvents on the oxidative solvolysis of lignin is still uncertain. Pan et al. [91] found that acetonitrile and methanol exhibit a positive effect on the oxidative degradation of lignin, but dioxane and ethanol exhibit a negative effect. It was reported that a composite-solvent composed of water and an organic solvent could both promote depolymerization of lignin and prevent repolymerization of lignin fragments (monomers), increasing the yield of the aromatic products [66].

In addition, under specific reaction conditions the solvent will play a different role as opposed to its normal role. Deng et al. [74] proposed that methanol as a solvent in oxidative solvolysis of lignin model compounds, which could afford active H species for the hydrogenolysis of the β-O-4 bond, producing phenol and acetophenone. Similarly, Shilpy et al. [92] investigated the influence of the property of the solvent on the oxidative solvolysis of vanillyl alcohol and vanillin. The highest conversion rate was observed in acetic acid (91%), followed by ethyl alcohol (70%), acetonitrile (67%), *N*,*N*-dimethyl formaldehyde (35%), tetrahydrofuran (20%) and acetone (1%). Thus it was proposed that the major role was played by peracetic acid that was in-situ generated by reacting acetic acid with H_2_O_2_ over an acid catalyst [93]. Then, peracetic acid accelerated and acted as an oxidant in the oxidation reaction.

Most of the solvents in the LDP process act as agents for dissolving lignin, products and homogeneous catalysts. In some cases, simple alcohols could exert an unexpected influence for protecting the produced aromatic fragments during the oxidative solvolysis process of lignin. The multi-function of solvents should be further investigated for the low-cost and high-efficient oxidative solvolysis of lignin.

## 4. Oxidative Solvolysis of Lignin-Related Model Compounds

The complexity and variability of lignin structure promoted the use of lignin-related model compounds for LDP studies, giving rise to a better understanding of the cracking mechanism via specific inter-unit linkage thus promoting the production of aromatic compounds. In this section, oxidative solvolysis of lignin-related model compounds (both aromatic monomers and oligomers) will be overviewed, concentrating on the reforming of aromatic monomers and the cracking of inter-unit linkages in oligomers.

### 4.1. Oxidative Reforming of Lignin-Derived Monomers

To understand the reactivity of the functional groups on lignin, much attention have been paid to the oxidative reforming of aromatic monomers. The representative lignin-related aromatic monomers such as apocynol, veratryl alcohol, 3-methoxy-4-hydroxybenzyl alcohol and 4-hydroxybenzyl alcohol were studied in recent years.

#### 4.1.1. Apocynol

**Figure 4:**
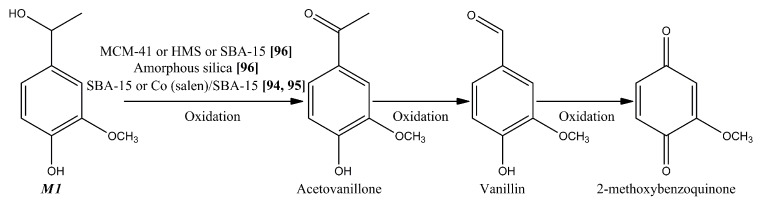
Reaction pathway for apocynol (***M1***) conversion on the catalysts.

Sushanta et al. [94,95] reported an efficient oxidative solvolysis of apocynol (***M1***) over SBA-15 and Co (salen)/SBA-15 by using H_2_O_2_ as an oxidant. The reaction pathway for ***M1*** conversion on SBA-15 and Co (salen)/SBA-15 is shown in Figure 4. The first oxidation product was acetovanillone, then vanillin was formed through the side chain cleavage while 2-methoxybenzoquinone as a product of oxidation of the phenolic group together with oxidative degradation of the *p*-alkyl side chain. Co (salen)/SBA-15 showed a highly efficient performance (nearly 100%) for ***M1*** conversion after 40 min microwave heating and SBA-15 showed an unusual oxidative ability and excellent selectivity for acetovanillone. Badamali et al. [96] also demonstrated the oxidation of ***M1*** by mesoporous MCM-41, HMS, SBA-15 and amorphous silica with using H_2_O_2_ as an oxidant under microwave irradiation. With the same conversion pathway on SBA-15 or Co (salen)/SBA-15, acetovanillone, vanillin and 2-methoxybenzoquinone were the main products (Figure 4). Silica as a catalyst led to the highest conversion (94%) of ***M1*** among the studied catalysts with the equal selectivity to acetovanillone and 2-methoxybenzoquinone. It could be concluded that the surface hydroxyl groups and internal pore structure of the employed silicas play a key role in the catalytic oxidative process. In addition, Hanson et al. [97] reported that dipicolinate vanadium complexes oxidized 2-phenoxyethanol in air. After the reaction at 100 °C for one week, approximately 20% of the 2-phenoxyethanol was consumed. Phenol (18%), formic acid (6%), and several other minor unidentified products were detected.

#### 4.1.2. Veratryl alcohol/3-methoxy-4-hydroxybenzyl alcohol/4-hydroxybenzyl alcohol

**Scheme 1:**
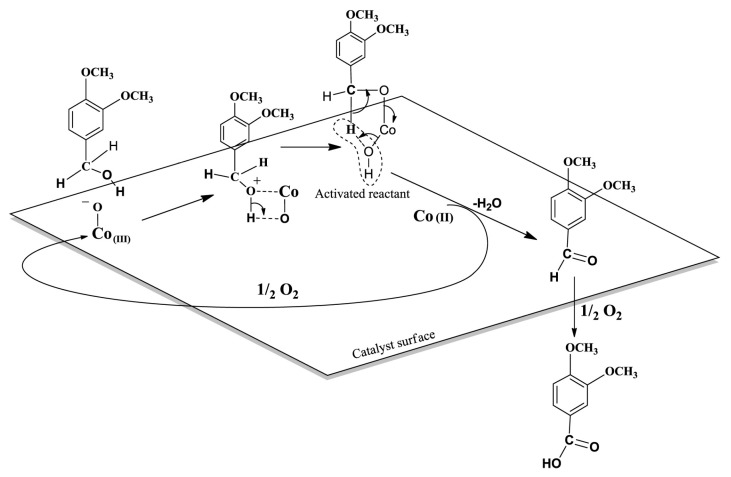
Proposed pathways for oxidation of veratryl alcohol over Co_3_O_4_ catalyst [99], reproduced with permission from Elsevier.

Mate et al. [98,99] reported the oxidation of veratryl alcohol over a nano-structured, spinel Co_3_O_4_ catalyst in the presence of O_2_. After 7 h of reaction at 140 °C in water, the Co_3_O_4_ catalyst yielded the highest conversion of 85% together with a 96% selectivity to veratryl aldehyde. In different solvents, the catalytic activity of the Co_3_O_4_ catalyst was proved to occur in the following order: toluene > water > ethanol > methanol. The highest conversion (75%) was observed in a nonpolar solvent such as toluene together with above 97% selectivity to veratryl aldehyde. The proposed pathway for oxidation of veratryl alcohol over Co_3_O_4_ catalyst was presented in Scheme 1: The adsorbed veratryl alcohol formed an intermolecular bond between Co^3+^ and O_2_ while the surface oxide ion formed an intermolecular bond with the hydrogen of the alcoholic group; then the abstraction of hydrogen by the oxide ion lead to the formation of Co^3+^OH species; after that the activated Co^3+^OH species could produce another intermolecular hydrogen bond and abstraction of an electron resulted in the formation of Co^2+^, H_2_O and the main product, veratryl aldehyde; the reduced Co^2+^ species were reoxidized with O_2_ to regenerate the active Co^3+^O^−^ species.

**Figure 5:**
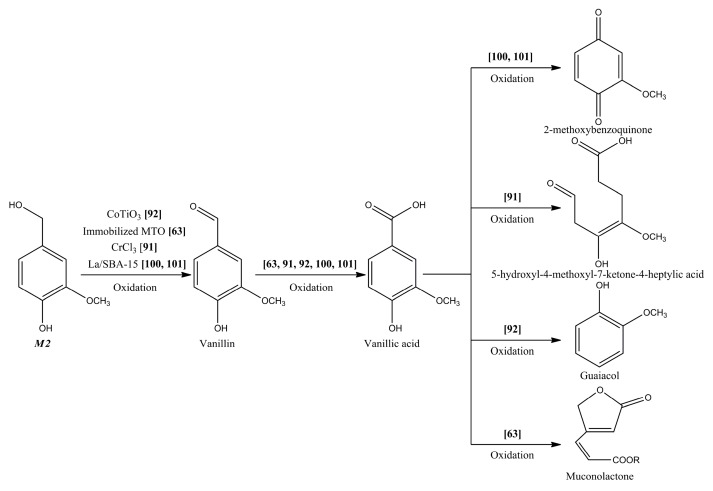
Reaction pathway for 3-methoxy-4-hydroxybenzyl alcohol (***M2***) conversion on the catalysts.

**Figure 6:**
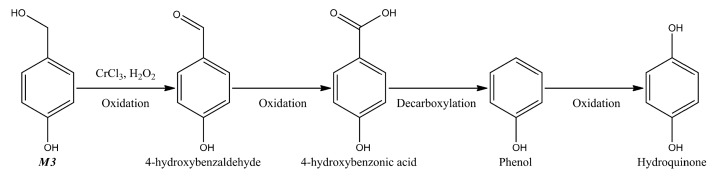
Reaction pathway for 4-hydroxybenzyl alcohol (***M3***) conversion on the catalyst [91], reproduced with permission from American Chemical Society.

**Scheme 2:**
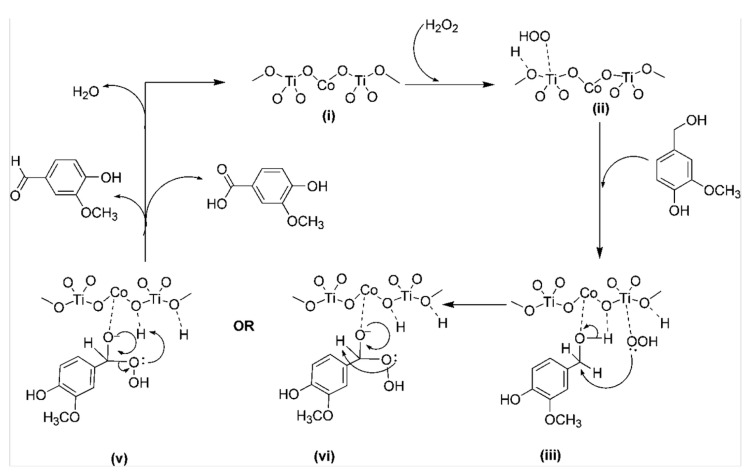
The suggested mechanism for formation of the main oxidation product (vanillin and vanillic acid) from vanillyl alcohol in the presence of CoTiO_3_ [92], reproduced with permission from Royal Society of Chemistry.

Gu et al. [100,101] performed the oxidation of a lignin model compound, 3-methoxy-4-hydroxybenzyl alcohol (***M2***), over a mesoporous La/SBA-15 catalyst. Sixty-eight percent conversion of ***M2*** to vanillin was obtained after 30 min of reaction under 200 W microwave irradiation with the presence of H_2_O_2_ as oxidant. Reaction pathway for ***M2*** conversion on La/SBA-15 was shown in Figure 5. Pan et al. [91] also reported the microwave-assisted oxidative degradation of 3-methoxy-4-hydroxybenzyl alcohol (***M2***) and 4-hydroxybenzyl alcohol (***M3***) with 14 types of metal salts in the presence of H_2_O_2_. After 10 min of reaction with a CrCl_3_ catalyst at 80 °C in H_2_O/acetonitrile (1:1, *v*/*v*), ***M2*** was converted to vanillin (59.8%), vanillic acid (9.7%), 5-hydroxyl-4-methoxyl-7-ketone-4-heptylic acid (17.0%), etc., through the pathway depicted in Figure 5. Different from the conversion on La/SBA-15, vanillic acid underwent a ring-opening reaction to produce 5-hydroxyl-4-methoxyl-7-ketone-4-heptylic acid. Under the same reaction conditions, ***M3*** was converted to hydroquinone (33.2%), 4-hydroxybenzaldehyde (22.2%), 4-hydroxybenzonic acid (7.6%), etc. The reaction pathway for ***M3*** conversion on CrCl_3_ was shown in Figure 6. 4-hydroxybenzaldehyde and 4-hydroxybenzonic acid were the first two oxidation products from ***M3***. Then the decarboxylation of 4-hydroxybenzonic acid resulted in the formation of phenol and then phenol reacted with hydroxyl radicals formed by a microwave irradiation of H_2_O_2_, to produce hydroquinone. In addition, the pathway for ***M2*** on immobilized MTO [63] and on CoTiO_3_ [92] was also different from the conversion on La/SBA-15 and on CrCl_3_ (Figure 5). After 6 h of reaction with immobilized MTO catalyst at room temperature in CH_3_COOH/H_2_O_2_, ***M2*** was converted to vanillin (14.2%), vanillic acid (18.9%), muconolactone (5.5%), etc. The muconolactone was mostly gained via an excessive oxidative ring opening reaction to a muconic acid intermediate followed by the formation of a lactone moiety, perhaps catalyzed by MTO. For the CoTiO_3_ catalyst, with the help of H_2_O_2_ in acetic acid and isopropanol solvent, a remarkable selectivity of 99.8% to vanillin was obtained at 85 °C. The major products were vanillin, vanillic acid and guaiacol. Scheme 2 illustrated the proposed mechanism for the oxidation of vanillyl alcohol over CoTiO_3_ [92]. Initially the catalyst reacted with H_2_O_2_ to form a hydroperoxyl radical, which acts as a source of highly active oxidant, and then attacks the catalyst to form an intermediate. When vanillyl alcohol was activated on the catalyst surface, the electron-rich hydroperoxyl attacks the partially positive charged carbon and the hydrogen of the hydroxyl group through oxygen to form an activated oxygen species. This facilitated the removal of the hydroxyl group from the hydroperoxyl and produced water as a by-product. At the same time, the vanilloxy cation released a proton that was attacked by electron-rich oxygen of the hydroperoxyl group to produce the final product, vanillic acid.

### 4.2. Oxidative Cracking of Typical Inter-Unit Linkages in Lignin-Derived Oligomers

#### 4.2.1. C–C Linkage

**Figure 7:**
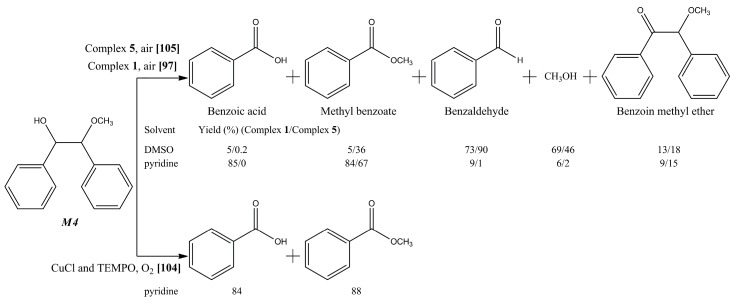
The aerobic oxidation of 1,2-diphenyl-2-methoxyethanol (***M4***) with vanadium and copper catalysts.

Homogeneous copper and vanadium complexes are well-known to be effective catalysts for aerobic oxidation reactions of diols with concomitant C–C bond cleavage [102,103]. Recognizing that copper and vanadium complexes could potentially be used for the C–C bond cleavage in lignin depolymerization, some scientists began to synthesize oxovanadium (e.g., Complex **1** in Figure 3) and copper catalysts and explore their fundamental reactivity. Hanson et al. [97,104] synthesized a series of vanadium complexes and homogeneous copper catalysts to explore their reactivity toward aerobic oxidation of a simple lignin model compound, 1,2-diphenyl-2-methoxyethanol (***M4***). The vanadium complex (dipic)V^V^(O)(O*^i^*Pr) (Complex **1**) reacted with ***M4*** in DMSO with the addition of air at 100 °C, producing predominantly benzaldehyde and methanol, but in pyridine it produced predominantly benzoic acid and methyl benzoate (Figure 7). The ketone benzoin methyl ether was an intermediate in this reaction. The CuCl/2,2,6,6-tetramethylpiperidine-1-oxyl radical (TEMPO) mixture could also react with ***M4*** in pyridine under O_2_ at 100 °C, affording a mixture of benzaldehyde (84%) and methyl benzoate (88%) (Figure 7). Unlike the vanadium catalyst system, the copper-catalyzed reactions were proposed for direct C–C bond cleavage of ***M4*** without ketone or aldehyde intermediates. Recently, four new vanadium(V) complexes of amino-bis(phenolate) ligands (e.g., Complex **5** in Figure 3) have been synthesized, and all complexes were employed for the catalytic aerobic oxidative C–C bond cleavage of ***M4*** in air at 100 °C, where benzaldehyde and methyl benzoate were identified as the major products [105]. In DMSO, a reaction with Complex **5** produced benzaldehyde at 90%, methanol at 46%, methyl benzoate at 36%, benzoin methyl ether at 18%, and a small amount of benzoic acid. In pyridine, methyl benzoate (67%), benzoin methyl ether (15%), and methanol (2%) were obtained. Benzaldehyde was detected as a minor product (1%) and no benzoic acid was detected.

**Figure 8:**
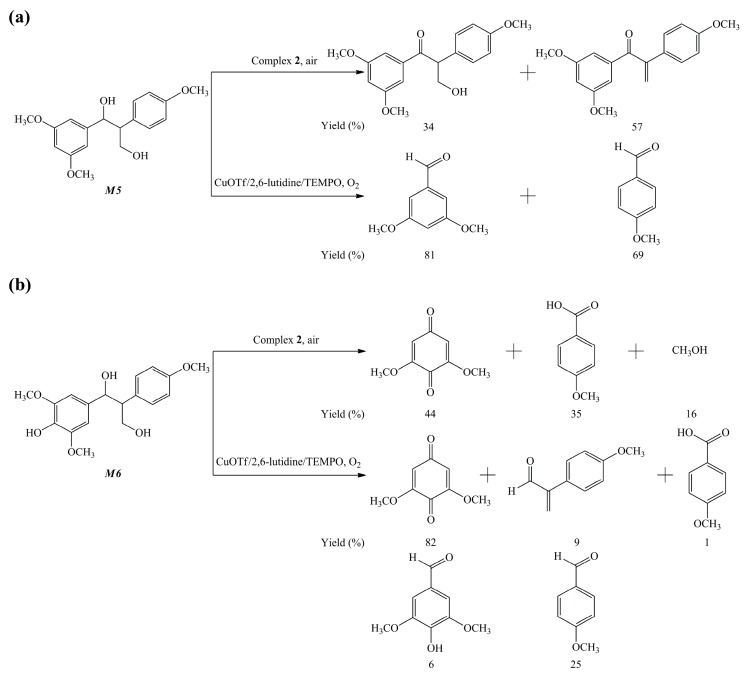
(**a**) The aerobic oxidation of non-phenolic β-1 linkage lignin model compound (***M5***) with vanadium and copper catalysts; and (**b**) the aerobic oxidation of phenolic β-1 linkage lignin model compound (***M6***) with vanadium and copper catalysts [104], reproduced with permission from American Chemical Society.

Degradation research indicated that β-1 structures are widespread, especially in hardwood lignin [106]. The β-1 linkage has not yet been adequately addressed by reductive cleavage. However, Hanson et al. [47,104,107] successfully used their CuOTf/2,6-lutidine/TEMPO catalyst system and (HQ)_2_V^V^(O)(O*^i^*Pr) (Complex **2**) to achieve the aerobic oxidation of nonphenolic and phenolic β-1 lignin models (***M5*** and ***M6***). It was found that the selectivity of the aerobic oxidation of β-1 lignin model compounds depended on both the catalyst and substrate. In the absence of phenolic groups, Complex **2** showed a high selectivity for C_α_–H bond cleavage, however the copper catalyst system worked on C_α_–C_β_ bond cleavage (Figure 8a). The introduction of a phenolic functional group in the substrate enabled the cracking of the C_α_–C_aryl_ bond with the help of both vanadium and copper catalysts (Figure 8b). As for the copper catalyst, the reaction of C–C bond cleavage might proceed via either a one-electron oxidation or a selective oxidation of the primary alcohol, followed by a retro-aldol reaction, while, for the vanadium catalyst, the reaction was in accordance with a two-electron oxidation pathway [47,104].

#### 4.2.2. C–O Linkage

The benzylic C–H or C–OH bonds in lignin would be easily attacked and transformed to carbonyl groups under oxidative conditions. Next, the C_α_–O or C_β_–O bond can be cracked to the corresponding fragments [3]. The oxidation of α-O-4 or β-O-4 linkages containing lignin model compounds has been extensively investigated. The key for achieving the production of specific chemicals from selective oxidation of lignin depends on the advance of catalysts.

#### 4.2.3. α-O-4 Containing Model Compound

**Figure 9:**
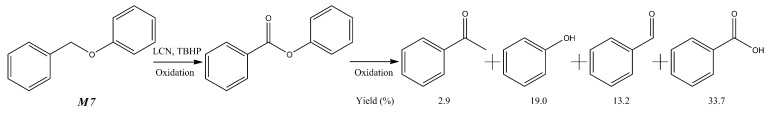
Reaction pathway for α-O-4 lignin model dimer (***M7***) conversion on the catalyst [64], reproduced with permission from John Wiley and Sons.

Haibach et al. [108] investigated the dehydroaryloxylation of aryl alkyl ethers in *p*-xylene using pincer iridium catalysts at a moderate temperature (150 or 200 °C). This system showed a rare atom-economical process for C–O ether bond cleavage, producing a limited number of substituted alkyl aryl ethers. Recently, Gao et al. [64] used nitrogen-containing graphene material (LCN) as an effective catalyst for the oxidation of α-O-4 types of a lignin model compound (***M7***) in the presence of *tert*-butyl hydroperoxide (TBHP), to produce aromatic aldehydes and acids in high yield. The reaction pathway for ***M7*** conversion on LCN is shown in Figure 9. A free-radical mechanism was involved, initiated by a benzylic C–H bond activation, followed by a C_α_–O bond cleavage, and finally completed by a further oxidation of intermediate aromatics. Similarly, an oxidative C–O bond cleavage of ***M7*** was demonstrated on Complex **5** at 80 °C, but the conversion was significantly limited [105].

#### 4.2.4. β-O-4 Containing Model Compound

**Figure 10:**
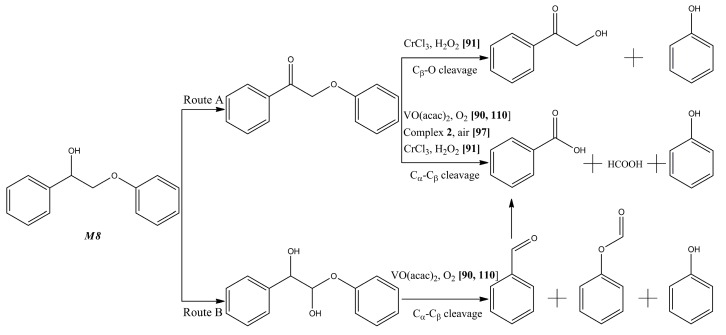
Reaction pathway for β-O-4 lignin model dimer (***M8***) conversion on the catalysts.

The oxidative solvolysis of a lignin model compound, 2-phenoxy-1-phenylethanol (***M8***), was performed with a CrCl_3_ catalyst while using H_2_O_2_ as an oxidant at 80 °C [91]. After 10 min of reaction in H_2_O/acetonitrile solvent, ***M8*** was converted to phenol (3.1%), benzoic acid (7.4%), 2-hydroxy-1-phenylethanone (17.3%), 2-phenoxy-1-phenylethanone (20.1%), etc. The reaction pathway for ***M8*** on CrCl_3_ is shown in Figure 10. ***M8*** was firstly oxidized to produce 2-phenoxy-1-phenylethanone. The β-O-4 bond in 2-phenoxy-1-phenylethanone was cleaved through two pathways: (1) between the C_β_–O atoms with 2-hydroxy-1-phenylethanone and phenol as the major product; and (2) between the C_α_–C_β_ atoms with benzoic acid as the major product. It is estimated that the cleavage of the C_β_–O bond was easier than that of the C_α_–C_β_ bond. Hanson et al. [109] used the CuOTf/2,6-lutidine/TEMPO catalyst system to conduct the aerobic oxidation of ***M8*** obtaining the overall conversion of 67% after 40 h at 100 °C. The major products were the formylated substrate 2-phenoxy-1-phenylformate, and the TEMPO-functionalized ketone 2-phenoxy-1-phenyl-2-(2,2,6,6-tetramethylpiperidin-1-yloxy)ethanone, while phenol, benzoic acid and 2-phenoxyacetophenone were produced only in small amounts. Recently, a new strategy for catalytic oxidation cleavage of aryl ethers in aralkyl aryl ethers involving a hemiacetal-like structure was proposed: VO(acac)_2_-catalyzed aerobic oxidation of ***M8*** in the presence of acetic acid [90,110]. Benzaldehyde (5%), benzoic acid (14%), phenyl formate (9%), phenol (54%), 2-phenoxy-1-phenylethanone (2%) and some esterification products (16%) were obtained after 8 h at 80 °C. It was proposed that a cleavage of the linkage (C–C bond and C–O bond) between the two aromatic rings could occur through at least two pathways: route A and a new route B (Figure 10). For route A, 2-phenoxy-1-phenylethanone was the intermediate that further cleaved to benzoic acid, phenyl formate and phenol. For route B, an initial oxidative activation of the C_β_–H bond produced a β-hydroxyl hemiacetal. Then, through a further cleavage of the C–C bond, the following oxidative activation of the C_α_–H bond, and the eventual cleavage of the C–O bond can generate benzaldehyde, benzoic acid, phenyl formate and phenol.

**Figure 11:**
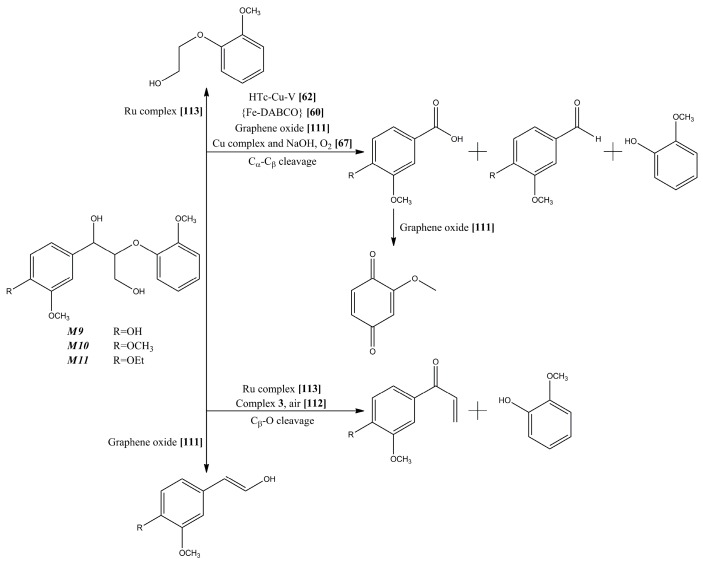
Reaction pathway for β-O-4 lignin model dimer (***M9***, ***M10***, and ***M11***) conversion on the catalysts.

The catalytic oxidative solvolysis of 1-(4-hydroxy-3-methoxyphenyl)-2-(2-methoxyphenoxy)-propane-1,3-diol (***M9***) and 1-(3,4-dimethoxyphenyl)-2-(2-methoxyphenoxy)-propane-1,3-diol (***M10***) was performed over a 2:1 mixture of 1,10-phenanthroline and copper (II) sulfate pentahydrate in NaOH solution [67]. This reaction was conducted in the presence of O_2_ in methanol at 80 °C. Vanillin was the major identified product from ***M9*** while veratric acid was the major product from ***M10*** (Figure 11). The β-O-4 bond was cleaved mainly between the C_α_–C_β_ atoms and different mechanisms were proposed because of the phenolic hydroxyl group *para* to the side chain. Blandez et al. [111] found that graphene oxide promoted the oxidative degradation of ***M9*** mainly to guaiacol, 2-methoxyquinone, vanillic acid and coniferyl aldehyde. After 24 h of reaction at 140 °C in acetonitrile solvent in the presence of O_2_, ***M9*** was converted to coniferyl aldehyde (20%), vanillic acid (2%), vanillin (6%), 2-methoxyquinone (5%) and guaiacol (96%). Reaction pathway for ***M9*** on graphene oxide was shown in Figure 11. {Fe-DABCO} oxidative cleavage of ***M10*** with peroxides in DMSO was also reported [60]. Guaiacol and veratraldehyde with 42% and 35% yield were obtained as the major products at 100 °C after 16 h. The reaction pathway for ***M10*** on {Fe-DABCO} is shown in Figure 11. Transition-metal-containing hydrotalcites (HTc) and V(acac)_3_/Cu(NO_3_)_2_·3H_2_O mixtures were employed for the catalytic cleavage of ***M10*** with O_2_ as an oxidant [62]. After 16 h of reaction at 135 °C in pyridine solvent in the presence of O_2_, ***M10*** was mainly converted to veratraldehyde (38%), veratric acid (31%) and phenol. The reaction pathway for ***M10*** on HTc is shown in Figure 11.

A ligand is an ion or molecule (functional group) linked to a central metal atom to structure a coordination complex. Compared to other ligands, salen complexes are relatively stable, inexpensive and can be readily synthesized. A series of vanadium (V) complexes (e.g., Complex **3** in Figure 3) bearing a Schiff base ligand for the non-oxidative solvolysis of a lignin model compound (***M11***) was demonstrated at 80 °C [112]. Different from the conversion pathway on copper complex, the β-O-4 bond was cleaved mainly at the C_β_–O bond (Figure 11). Thus ***M11*** was converted to alkene, 2-methoxyphenol and ketone after 24 h of reaction in CD_3_CN solvent. Another series of dipicolinate vanadium (V) complexes (e.g., Complex **2** in Figure 3) for the oxidative degradation of 1-phenyl-2-phenoxyethanol (***M8***) was studied at 100 °C in air [97]. Differently, ***M8*** was mainly converted to formic acid, benzoic acid, phenol, and 2-phenoxyacetophenone (Figure 10). Ruthenium-triphos complexes also exhibited an outstanding catalytic activity and selectivity in the redox-neutral C–C bond cleavage of ***M10*** [113]. The use of Ru(CO)(Cl)(H)–(triphos) [114] resulted in the products from the C_α_–C_β_ bond cleavage in 66% yield, whereas the products from the C_β_–O bond cleavage were formed in 4% yield at 160 °C after 4 h of reaction (Figure 11). It should be noted that the phenolic group played an important role: (1) C_α_–OH was required for both the C_α_–C_β_ and the C_β_–O bond cleavage; and (2) C_γ_–OH was essential for accessing to the C_α_–C_β_ bond cleavage.

**Figure 12:**
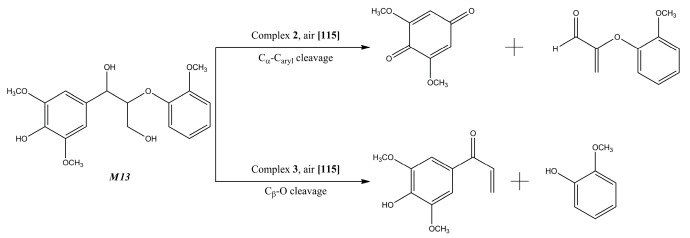
Reaction pathway for β-O-4 lignin model dimer (***M13***) conversion on the catalysts.

To compare the differences in selectivity of products between Complex **2** and Complex **3**, the aerobic oxidation of a lignin model compound (***M13***) reacting with Complex **2** and Complex **3** was investigated [115]. Complex **3** afforded the production of alkene, 2-methoxyphenol and ketone from C_β_–O bond cleavage, while Complex **2** gave the production of 2,6-dimethoxybenzoquinone and acrolein derivative, and ketone from a C_α_–C_aryl_ bond cleavage (Figure 12). An application of an isotope tracer technique showed that Complex **3** broke the C–O bond in the model compound via cracking of the benzylic C_α_–H bond, while Complex **2** broke the C_α_–C_aryl_ bond without cleavage of the benzylic C_α_–H bond. Further experiments showed that no C_α_–C_aryl_ bond cleavage was observed in the aerobic oxidation of a non-phenolic model compound (***M12***) with Complex **2**. It was consistent with the result of aerobic oxidation of a β-1 linkage lignin model compound on Complex **2** (Figure 8). Mechanisms of Complex **2** and Complex **3** catalyses during reactions were proposed in a recent publication [116]. Parker et al. [117] attempted to determine the effect of ligand structure on the activity of vanadium Schiff-base catalysts (e.g., Complex **3**) towards the degradation of β-O-4 contained dimers. *Ortho*-bulky, electron-donating ligand substituents were determined to make the most active catalyst, which can be enhanced by the addition of bulky aliphatic substituents such as *tert*-butyl and adamantyl groups. The trityl-substituted complex was not that active due to the enhanced steric hindrance, confining the substrate access to the active sites of catalyst [117].

**Scheme 3:**
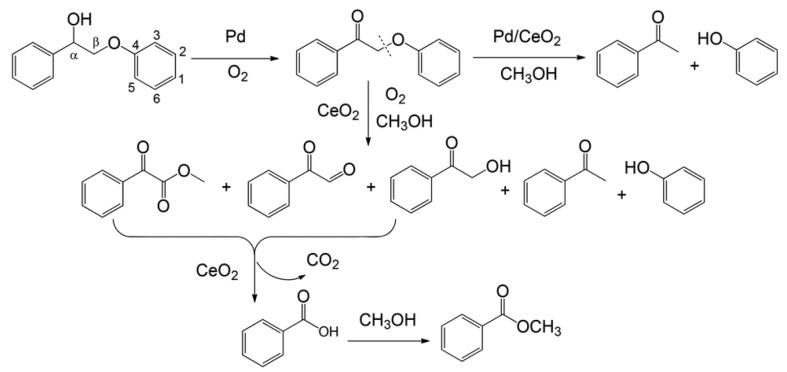
Proposed pathway for oxidative conversion of 2-phenoxy-1-phenylethanol (***M8***) over a Pd/CeO_2_ catalyst [74], reproduced with permission from Royal Society of Chemistry.

The oxidation of lignin-related model compounds, 2-phenoxy-1-phenylethanol (***M8***) and 2-(4-methoxyphenoxy)-1-phenylethanol (***M14***), on a Pd/CeO_2_ catalyst was performed in methanol under O_2_ [74]. The reaction mechanism for the conversion of ***M8*** over Pd/CeO_2_ in methanol with the presence of O_2_ is shown in Scheme 3. The C_α_–OH of 2-phenoxy-1-phenylethanol was first oxidized due to the catalytic function of Pd nanoparticles, to form 2-phenoxy-1-phenylethanone, whose β-O-4 bond became weakened due to the presence of C_α_=O. The subsequent hydrogenolysis of the β-O-4 bond of 2-phenoxy-1-phenylethanone resulted in the form of acetophenone and phenol over the Pd/CeO_2_ catalyst. On the other hand, the oxidative cracking of the β-O-4 bond over CeO_2_ might lead to the formation of phenol and an oxidized intermediate (e.g., 2-hydroxyacetophenone). Meanwhile, a small amount of acetophenone can be produced during this process. Finally, the oxidized intermediate experienced the C–C bond cracking, producing benzoic acid and methyl benzoate. Phenol, acetophenone and methyl benzoate were produced with the yield of 48%, 38% and 14% from the oxidation of ***M8***, while 4-methoxyphenol, acetophenone and methyl benzoate were produced with yields of 82%, 38% and 40% from the oxidation of ***M14*** at 185 °C for 24 h. It could be concluded that *ortho*-methoxy substituents further elevated the performance of the Pd/CeO_2_ catalyst, which was consistent with the other report stating that *ortho*-methoxy substituents could weaken the stability of the aromatic ring, promoting its over-oxidation [64].

**Scheme 4:**
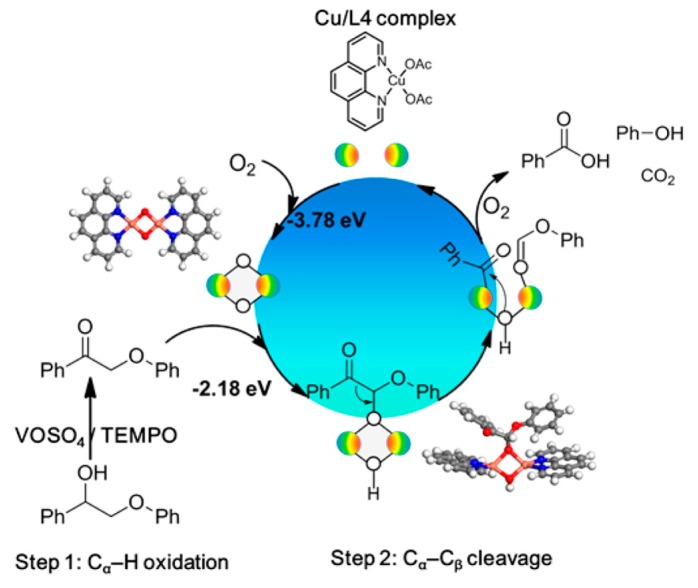
Proposed reaction mechanism for two-step, catalytic C–C bond oxidative cleavage process [118], reproduced with permission from American Chemical Society.

Moreover, Wang et al. [118] reported a two-step strategy for lignin C–C bond conversion via a β-O-4 alcohol oxidation to ketone over the VOSO_4_/TEMPO [(2,2,6,6-tetramethylpiperidin-1-yl)oxyl)] catalyst, followed by a ketone oxidation over Cu(OAc)_2_/1,10-phenanthroline catalyst to acids and phenols. The proposed reaction mechanism is shown in Scheme 4. The oxidation of a C_α_–OH alcohol to a ketone activated the C_β_–H bond. The Cu(OAc)_2_/1,10-phenanthroline reacted with oxygen to form a copper-oxo-bridged dimer. The activation of the C_α_-C_β_ bond in the form of a hydroxyl ketone structure-like intermediate significantly decreased its bond energy, resulting in the formation of benzoic acid and phenyl formate. Transfer of a hydroxyl group generated benzoic acid, restoring the initial copper complex. Finally, the oxidative decarboxylation of phenyl formate generated phenol and CO_2_.

The oxidative solvolysis of lignin-related model compounds with homogeneous catalysts was mostly reported for enhancing the production of aromatic aldehydes, aromatic ketones, benzoquinones, carboxylic acids, etc. The oxidative solvolysis of model compounds with heterogeneous catalysts may achieve a high selectivity of specific products. However, the performance of both homogeneous and heterogeneous catalysts in the cleavage of inter-unit linkages is still ambiguous in the oxidative solvent system, confining the understanding of inherent mechanism of whole lignin depolymerization process.

## 5. Summary and Outlook

The catalytic oxidation of lignin in solvent systems is attracting more and more attention for producing renewable aromatic chemicals. A number of functionalized monomeric products were obtained from oxidative depolymerization of lignin, including vanillin, vanillic acid, syringaldehyde and syringic acid. Considering the ambiguities in the inherent mechanism of (catalytic) oxidative solvolysis of lignin process and the limited selectivity and yield of renewable chemicals, several issues should be addressed: (1) identification of the oligomer structure for specifying the inherent oxidative depolymerization mechanism of natural lignin; (2) design of highly efficient catalyst for C–C type inter-unit linkages; (3) strategies for suppressing the repolymerization of the produced fragments; (4) low-cost separation and purification technology of the specific aromatic compounds from an LDP system; and (5) stability of the heterogeneous catalysts during LDP and its reactivation.

## Figures and Tables

**Table 1:** Summary of oxidative depolymerization of the raw lignin material.

Entry	Feedstock	Conditions	Solvent	Catalyst	Products	Yield	Ref.
1	Organosolv lignin (from *M.* giganteus)	80 °C, 24 h, air	Acetonitrile-THF or ethyl acetate-THF	Vanadium complexes bearing Schiff base ligands	Monophenolic compounds (vanillin, syringic acid, syringaldehyde)	0.78 wt %, 0.67 wt %, 0.59 wt % (Catalyst = Complex **3**)	[58]
2	Organosolv lignin	100 °C, 18 h, 0.8 MPa synthetic air	Ethyl acetate	Vanadium complexes and other organometallic catalysts	Bio-oil	*M*w = 575 Da (Catalyst = Complex **2**)	[59]
3	Organosolv lignin and Kraft lignin	100 °C, 8 h, H_2_O_2_	DMSO and acetic acid	{Fe-DABCO}	Bio-oil	/	[60]
4	Organosolv lignin	80 °C, 24 h, air	Acetonitrile-THF	Co(salen) supported on graphene oxide	Vanillin (main)	3067 g	[61]
5	Organosolv lignin and Kraft lignin	135 °C, 40 h, 1.0 MPa O_2_	Pyridine	V(acac)_3_ and Cu(NO_3_)_2_·3H_2_O or HTc-Cu-V	Bio-oil	*M*w = 300 Da	[62]
6	Hydrolytic sugar cane lignin and red spruce kraft lignin	25 °C, 24 h, H_2_O_2_	Acetic acid	MTO or poly(4-vinylpyridine)/MTO or polystyrene/MTO	Bio-oil	/	[63]
7	Organosolv ligin (from birch wood)	140 °C, 24 h, 0.1 MPa O_2_	H_2_O, *tert*-butyl hydroperoxide (TBHP)	Nitrogen-containing graphene material (LCN)	Bio-oil	45.8 wt %	[64]
8	Alkali lignin	175–225 °C, 0–1 h, 0.5–1.5 MPa O_2_	Water	NaOH	Formic acid, acetic acid, succinic acid, oxalic acid, glutaconic acid	44.0 wt %	[65]
9	Wheat alkali lignin	150 °C, 1 h, H_2_O_2_	Water/methanol/1,4-dioxane/tetrahydrofuran/ethanol	CuO, Fe_2_(SO_4_)_3_ and NaOH	Monophenolic compounds	17.92 wt % (in methanol-water)	[66]
10	Wood lignin from Loblolly pine	80 °C, 24 h, 0.27/1.24 MPa O_2_	Methanol	Copper-phenanthroline complex and NaOH	Vanillic acid, vanillin	3.5 wt %, 12.6 wt %	[67]
11	Kraft lignin	170 °C, 0.3 h, 0.5 MPa O_2_	Methanol-water and H_2_SO_4_	H_3_PMo_12_O_40_	Vanillin, methyl vanillate	5.2 wt %	[68]
12	Kraft lignin	170 °C, 0.3 h, 1.0 MPa O_2_	Methanol-water and H_2_SO_4_	H_3_PMo_12_O_40_	Vanillin, methyl vanillate	4.6 wt %, 4.2 wt %	[69]
13	Kraft lignin	170 °C, 1 h, 1.0 MPa O_2_	Methanol-water and H_2_SO_4_	CuSO_4_; FeCl_3_;	Vanillin, methyl vanillate	6.3 wt %	[70]
				CuCl_2_; CoCl_2_			
14	Kraft lignin	45 °C, 1 h, H_2_O_2_	Acetone-water	Metal salt catalysts	Vanillin-based monomers	0.51 wt %	[71]
15	Organosolv lignin	180 °C, 2 h, 13.8 MPa air	Acetic Acid-water	Co/Mn/Zr/Br mixture	Vanillin, vanillin acid, syringaldehyde, syringic acid	0.99 wt %, 2.91 wt %, 2.52 wt %, 4.51 wt %	[72]
16	Organosolv beech wood lignin	200W, 5–30 min, H_2_O_2_	NaOH solution	La/SBA-15	Vanillin, syringaldehyde	9.94 wt %, 15.66 wt %.	[73]
17	Organosolv lignin	185 °C, 24 h, 0.1 MPa O_2_	Methanol	Pd/CeO_2_	Vanillin, guaiacol, 4-hydroxybenzaldehyde	5.2 wt %, 0.87 wt %, 2.4 wt %	[74]
18	Enzymatic hydrolysis lignin	120 °C, 0–3 h, 0.5 MPa O_2_	NaOH solution	LaMnO_3_	Vanillin, *p*-hydroxybenzyl aldehyde, syringaldehyde	4.32 wt %, 2.03 wt %, 9.33 wt %	[75]
19	Enzymatic hydrolysis lignin	120 °C, 0–3 h, 0.5 MPa O_2_	NaOH solution	LaCoO_3_	Vanillin, *p*-hydroxybenzyl aldehyde, syringaldehyde	4.55 wt %, 2.23 wt %, 9.99 wt %	[76]
